# Enhancement of active compound, genipin, from Gardeniae Fructus using immobilized glycosyl hydrolase family 3 β-glucosidase from *Lactobacillus antri*

**DOI:** 10.1186/s13568-017-0360-y

**Published:** 2017-03-16

**Authors:** Young Soo Kim, Chung-Jo Lee, Jin Yeul Ma

**Affiliations:** Korean Medicine Application Center, Korea Institute of Oriental Medicine, Cheomdan-ro 70, Dong-gu, Daegu, 41062 Republic of Korea

**Keywords:** Glycosyl hydrolase family 3 β-glucosidase, Gardeniae Fructus, Geniposide, Genipin, *Lactobacillus antri*, Enzyme immobilization

## Abstract

Geniposide is an iridoid glycoside, which is abundant in Gardeniae Fructus. Despite the various pharmaceutical effects of geniposide on a human body, its hydrolysis into a smaller molecule, genipin, by β-glucosidase produced by bacteria in the intestines is particularly important to improve geniposide uptake into the body. Since geniposide is much more abundant in Gardeniae Fructus than its aglycone genipin, we herein transformed geniposide into genipin using purified recombinant β-glucosidase from *Lactobacillus antri* (rBGLa), which was expressed in *Escherichia coli* to enhance the genipin content. Purified rBGLa was characterized using *p*-nitrophenyl β-d-glucopyranoside, and the optimal temperature and pH for its β-glucosidase activity were found to be 45 °C and 6.0. When the enzyme was immobilized, rBGLa was active at higher temperatures than the free enzyme, and we confirmed that its stability upon changes in pH and temperature was highly improved. Using 0.5 μg/mL free rBGLa, single compound of 0.4 mM geniposide was efficiently converted into genipin within 2 h, and the immobilized rBGLa also successfully transformed geniposide in a hot-water extract of Gardeniae Fructus into the aglycone, which makes it applicable to the food and pharmaceutical industries.

## Introduction

Orally administered herbal medicine containing flavonoid components is generally absorbed into the blood vessels from the gastrointestinal tract and shows various effects on the human body (Kumar and Pandey 2013). In this process, the glycoside components containing the sugar structure are degraded into the non-glycosylated aglycone form by intestinal bacteria in the gastrointestinal tract, and the degraded smaller molecules of aglycones improve the absorption rate of these glycosides and exhibit higher efficacy than the glycone form (Hollman et al. 1999; Thilakarathna and Rupasinghe 2013; Yim et al. 2004). Intestinal bacteria have various glycoside hydrolase families including β-glucosidase, which are responsible for decomposing substrates having various sugar moieties according to each kind (Ioku et al. 1998; Nemeth et al. 2003). Lactic acid bacteria, especially *Lactobacillus*, are well-known probiotic bacteria present in the intestines that have been studied regarding probiotic applications, including food fermentation and deglycosylation of plant-derived glycosides using β-glucosidase from *Lactobacillus* (Ku et al. 2016; Urso et al. 2006; Xiudong et al. 2016).

Gardeniae Fructus is the dried mature fruit of *Gardenia jasminoides* Ellis that has been used as a traditional medicine for anti-inflammatory (Koo et al. 2006), hepatoprotective (Kim et al. 2013), antioxidant (Pham et al. 2000) and anti-angiogenic effects (Koo et al. 2004a). Geniposide, which is one of the iridoid glycosides, is a major component of Gardeniae Fructus, and when it is orally administered, it is hydrolyzed into the aglycone genipin by β-glucosidase in intestinal bacteria. After absorption into the human body, geniposide and genipin exhibit various pharmaceutical activities, including anti-inflammatory (Koo et al. 2004b), antioxidant (Wang et al. 2015), choleretic (Shoda et al. 2004), and anti-cancer effects (Kim et al. 2005). Despite these various effects of genipin, Gardeniae Fructus contains it at a very low concentration, whereas it has abundant geniposide, the β-glycoside-linked compound from which genipin is derived (Gong et al. 2014). A study in which the homogenate did not degrade geniposide despite containing β-glucosidase in rat liver suggested that the hydrolysis of geniposide relies on the β-glucosidase of intestinal bacteria (Akao et al. 1994). Therefore, to improve the genipin content in Gardeniae Fructus, an enzyme-catalyzed approach converting geniposide into genipin could be very useful.

In this study, we cloned and expressed recombinant β-glucosidase (EC 3.2.1.21) from *Lactobacillus antri* (rBGLa) belonging to glycoside hydrolase family 3 in *Escherichia coli* (*E. coli*), following the improvement of rBGLa activity and stability by enzyme immobilization in calcium-alginate carrier. Using the immobilized rBGLa, the geniposide in Gardeniae Fructus extract was efficiently hydrolyzed into genipin, which indicates the potential of rBGLa for industrial applications to produce valuable aglycones by transforming plant-derived glycosides.

## Materials and methods

### Hot-water extraction of Gardeniae Fructus

Gardeniae Fructus, a dried mature fruit of *Gardenia jasminoides* Ellis, was purchased from Yeongcheon Oriental Herbal Market (Yeongcheon, Republic of Korea) and deposited in the herb bank of the Korean Institute of Oriental Medicine. An extract was prepared by extracting 50 g of Gardeniae Fructus in 1000 mL of distilled water at 115 °C for 3 h (Gyeongseo Extractor Cosmos-600, Gyeongseo, Republic of Korea). After filtration through testing sieves (150 μm; Retsch, Germany), Gardeniae Fructus extract was freeze-dried and kept in a desiccator at 4 °C. The dried extract powder was stored at −20 °C until use.

### Bacterial strains, plasmid, and gene cloning

The glycosyl hydrolase family 3 β-glucosidase gene was amplified by polymerase chain reaction (PCR) with KOD FX-Neo DNA polymerase (Toyobo, Osaka, Japan), genomic DNA of *L. antri* DSM 16041 as a template, and the following primers (Cosmogentech, Republic of Korea): forward (5′-ACC ACA GCC AGG ATC CGA TGG TAA AAA TTG ATT TGC AG-3′) and reverse (5′-TTG AGA TCT GCC ATA TGT TAC TTT AAG GCC CGC AGA AA-3′) primers. The amplified PCR product was introduced into the pETDuet-1 vector (Novagen, USA) between *Bam*HI and *Nde*I sites by using the In-Fusion HD Cloning Kit (Takara, Kusatsu, Japan), and the resulting plasmid, named pET-BGLa, was transformed into *E. coli* BL21(DE3) (Novagen).

### Enzyme expression and purification of recombinant β-glucosidase

*Escherichia coli* BL21(DE3) harboring pET-BGLa was cultivated at 25 °C in Luria–Bertani medium containing 50 μg/mL ampicillin until OD_600_ reached 0.5 to express rBGLa by adding 0.2 mM isopropyl-β-d-thiogalactopyranoside (IPTG) and was additionally incubated for 8 h at the same temperature. The culture was harvested by centrifugation at 4000*g* for 20 min, and then washed with buffer containing 50 mM Na_2_HPO_4_ and 0.3 M NaCl (pH 7.0). Cells were then resuspended in the same buffer and disrupted by ultrasonication (Omni-Ruptor 4000; Omni International, Kennesaw, GA, USA). Cell debris was removed by centrifugation at 14,000*g* and 4 °C for 30 min. After the binding to TALON metal affinity resins (Takara), non-specific binding proteins were removed by washing with a buffer containing 50 mM Na_2_HPO_4_, 0.3 M NaCl, and 30 mM imidazole (pH 7.0). Finally, (His)_6_-tagged rBGLa was purified by the elution buffer containing 50 mM Na_2_HPO_4_, 0.3 M NaCl, and 250 mM imidazole (pH 7.0), which was confirmed by 12% sodium dodecyl sulfate polyacrylamide gel electrophoresis (SDS-PAGE).

### Enzyme characterization

The specific activity of free and immobilized rBGLa was evaluated using *p*NPG as a substrate in 25 mM sodium phosphate buffer (pH 6.0) at 45 °C. The *p*-nitrophenol (*p*NP) liberated by BGLa was measured immediately at 405 nm using a microplate reader (GloMax-Multi Microplate Multimode Reader; Promega, Madison, WI, USA). Specific activity was defined as μmol *p*NP generated/min/mg BGLa. The optimum temperature of BGLa was studied in 25 mM sodium phosphate buffer (pH 6.0) containing 5 mM *p*NPG at various temperatures ranging from 25 to 60 °C, and the thermal stability was determined by measuring residual BGLa activity after 2 h of incubation at pH 6.0 and each temperature. The effect of pH on BGLa activity at the optimum temperature was examined in the same buffer at various pH levels in the range from 3.0 to 10.0. The pH stability was evaluated after 2 h of incubation at optimum temperature and each pH. The stability of immobilized enzyme was assayed after washing the enzyme beads with the reaction buffer after 2 h of incubation.

The effects of cations and chemical reagents on rBGLa activity were investigated. rBGLa activity was evaluated at 45 °C in 25 mM Na_2_HPO_4_ (pH 6.0) additionally containing 10 mM NaCl, KCl, MgCl_2_, CaCl_2_, sodium dodecyl sulfate (SDS), Triton X-100, glycerol, imidazole. The effects were expressed as relative activities compared to that of rBGLa in the absence of cations and chemical reagents.

### Immobilization of rBGLa

Purified rBGLa was immobilized by the method using alginate carrier (Busto et al. 1995). rBGLa solution was mixed with 3% sodium alginate, then the rBGLa-alginate mixture was added dropwise into 0.3 M CaCl_2_ with continuous stirring at room temperature. Immobilized gel beads formed were cured for 2 h. After the gel beads were washed with 0.03 M CaCl_2_ solution, they were stored at 4 °C in 25 mM Na_2_HPO_4_ solution (pH 6.0).

### Enzymatic conversion of geniposide and hot-water extract of Gardeniae Fructus

Biotransformation of geniposide was performed by reacting 0.5 mM geniposide with 0.5 μg/mL BGLa enzyme at 45 °C in 25 mM sodium phosphate buffer (pH 6.0). Samples for High-performance liquid chromatography (HPLC) analysis were prepared by withdrawing them at regular intervals for 4 h. Freeze-dried hot-water extract of Gardeniae Fructus was dissolved in 25 mM sodium phosphate buffer (pH 6.0) to a concentration of 2 mg/mL, and an equal volume of 100 μg/mL free BGLa solution in the same buffer or appropriate amount of immobilized rBGLa was added to Gardeniae Fructus extract solution. Samples were also withdrawn during 4-h reaction hourly.

### HPLC analysis

Biotransformation samples with a volume of 10 μL, with both geniposide and Gardeniae Fructus extract, were analyzed by HPLC (CM5000; Hitachi, Tokyo, Japan) using a Geminin C18 column (5 µm, 250 × 4.6 mm; Phenomenex, Torrance, CA, USA) under an oven temperature of 40 °C. A mobile phase containing 1% (v/v) acetic acid in water (A) and acetonitrile (B) was used with the following gradient at a flow rate of 1.0 mL/min: (A) 85–35% (0–40 min), 35–0% (40–45 min), 0% (45–50 min), 0–85% (50–55 min), and 85% (55–70 min). The bioconversion of geniposide and Gardeniae Fructus extract was detected at a UV wavelength of 240 nm. Geniposide (Wako Pure Chemical Industries, Ltd., Osaka, Japan) and genipin (Sigma, St. Louis, MO, USA) were used as standards.

## Results

### Expression and purification of rBGLa

**Fig. 1:**
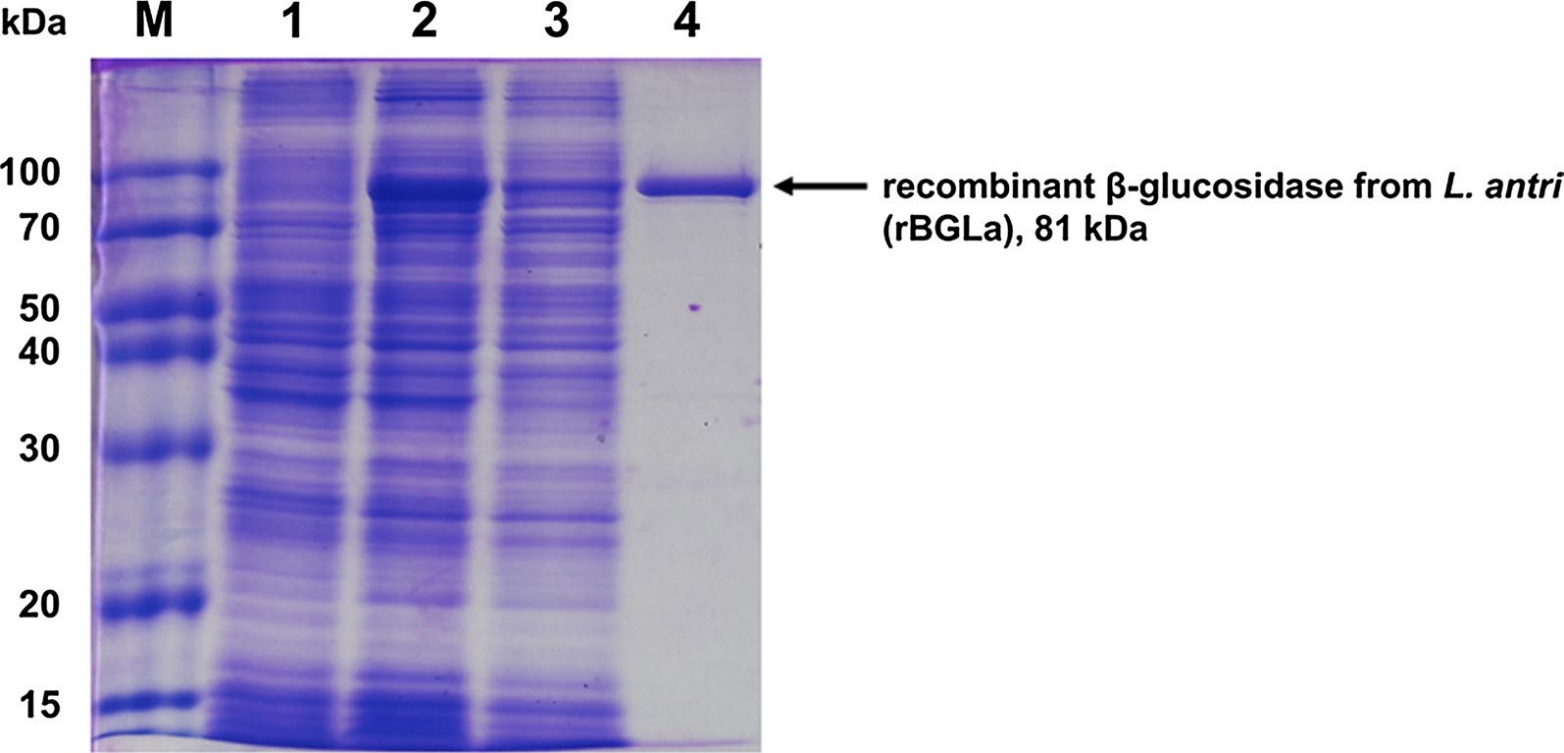
SDS-PAGE analysis through the procedure of (His)_6_-tag purification*. Lane M* molecular weight markers; *Lane 1* total soluble fraction of non-induced cell lysate; *Lane 2* total soluble fraction of IPTG-induced cell lysate; *Lane 3* the fraction passed through resin; *Lane 4* the fraction with elution buffer containing 250 mM imidazole

The β-glucosidase gene from *L. antri* DSM 16041 which is homologous to glycosyl hydrolase family 3 consists of 2208 bp and encodes 735 amino acids (UniProtKB accession number: C8P9L9). The gene was inserted into pETDuet-1 vector between the *Bam*HI and *Nde*I sites, which is located on the downstream of nucleotides encoding the (His)_6_-tag for affinity purification. The rBGLa was expressed by induction with 0.2 mM IPTG and at a low temperature, 25 °C, to improve the solubility of the target protein. After cell disruption, rBGLa was bound to the (His)_6_-tag affinity resin by applying the cell lysate into the column, and washed with buffer containing 30 mM imidazole to remove the non-specific binding proteins in *E. coli*. rBGLa was finally eluted in the buffer containing 250 mM imidazole. Throughout the purification process, the results exhibited 1.8-fold increase of rBGLa purity and 33.5% recovery yield (Table 1). The molecular weight of rBGLa, predicted to be approximately 81.0 kDa, was confirmed by SDS-PAGE (Fig. 1).

**Table 1:** Purification process of rBGLa

Purification steps	Total activity (U, μmol/min)	Total protein (mg)	Specific activity (U/mg)	Purification (fold)	Recovery (%)
Crude cell lysate (soluble)	4708	122.3	38.5	1	100
Affinity chromatography	1578	11.1	142.0	3.7	33.5

### Enzymatic characterization of free rBGLa

**Fig. 2:**
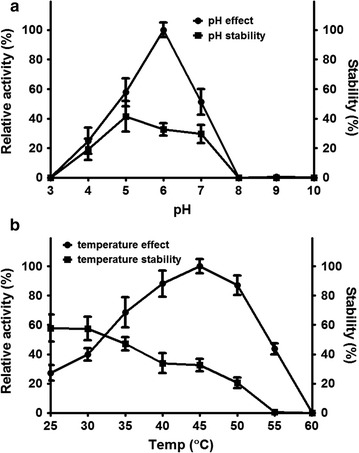
Effects of pH (**a**) and temperature (**b**) on the the activity and stability of free rBGLa. The enzyme activity was examined using *p*NPG as a substrate. Reaction samples were withdrawn at regular intervals, and OD_405_ was measured immediately using a microplate reader. The effects of pH and temperature were investigated at 45 °C for various pHs between 3.0 and 10.0. The effect of temperature (25–60 °C) was investigated in the reaction buffer (pH 6.0). The stability was calculated as the ratio of residual activity after 2 h of incubation to the initial activity

The effects of pH and temperature on β-glucosidase were investigated (Fig. 2). Purified rBGLa was active over the pH range of 4.0–7.0 at 45 °C, and in particular had more than 50% of its maximum activity at a moderately acidic pH of between 5.0 and 7.0 (Fig. 2a). The optimal pH of rBGLa activity was 6.0 in 25 mM sodium phosphate buffer, which is close to the pH at which *Lactobacillus* grows optimally (pH 6.2–6.5). The enzyme activity was always more than 50% of its maximum level between pH 5.0 and pH 7.0; however, there was no activity at pH 3 or at alkaline pH levels of more than 8.0. After 4 h of incubation over a pH between 3.0 and 10.0 at 45 °C, the result revealed that free rBGLa has low pH stability in the overall pH range, which exhibited 42% of the original activity at pH 5.0 (Fig. 2a).

rBGLa showed β-glucosidase activity over the temperature range of 25–55 °C at pH 6.0, and the optimal temperature for this activity was 45 °C (Fig. 2b). The enzyme also had more than 85% activity over the temperature range of 40–50 °C and less than 50% activity at temperatures below 30 °C and above 55 °C compared with the maximum activity. No enzyme activity was observed at 60 °C. Thermal stability of free rBGLa exhibited a maximum at 25 °C, which was 57.8% of the original activity. As the temperature rose, the thermal stability gradually decreased, which was completely lost above 55 °C.

The effect of the cations and chemical reagents on the BGLa activity was investigated (Table 2). rBLGa activity decreases in the presence of all cations examined at 10 mM. The activity was inhibited about 18% by monovalent cations (Na^+^ and K^+^), whereas the enzyme inhibition was increased in the presence of the divalent cations (Mg^2+^ and Ca^2+^), especially 89.6% of inhibition was observed on Ca^2+^. On the detergents having different properties the activity of the rBGLa was investigated. Enzyme activity was inactivated in the reaction buffer containing 10 mM SDS (99.4% inhibition), whereas the activity was preserved well in the presence of 10 mM Triton X-100. In addition, the imidazole used in the purification process inhibited 37.5% of rBGLa activity, which is similar to Mg^2+^ (31.7%).

**Table 2:** Effect of cations and chemical reagents on rBGLa activity

Cation or chemical reagent	Relative activity (%)
Cations	
Control	100.0
Na^+^	81.7
K^+^	82.4
Mg^2+^	68.3
Ca^2+^	10.4
Chemical reagents	
SDS	0.6
Triton X-100	93.9
Glycerol	90.1
Imidazole	62.5

### Enzymatic characterization of immobilized rBGLa

**Fig. 3:**
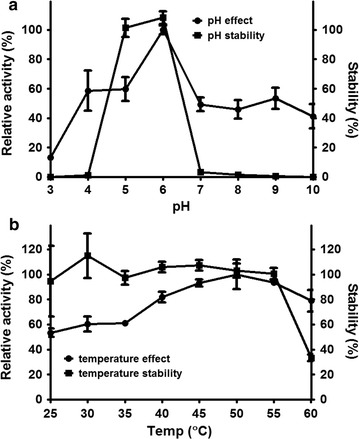
Effects of pH (**a**) and temperature (**b**) on the activity and stability of immobilized rBGLa. The enzyme activity was examined using *p*NPG as a substrate. Reaction samples were withdrawn at regular intervals, and OD_405_ was measured immediately using a microplate reader. The effects of pH and temperature were investigated at 45 °C for various pHs between 3.0 and 10.0. The effect of temperature (25–60 °C) was investigated in the reaction buffer (pH 6.0). The stability was calculated as the ratio of residual activity after 2 h of incubation to the initial activity

Purified rBGLa was entrapped using the cross-linking method in calcium alginate. The effects of pH and temperature on this immobilized rBGLa were investigated (Fig. 3). In the pH range of 5.0–7.0, the immobilized rBGLa displayed an activity pattern similar to that of the free enzyme, which also has maximum activity at pH 6.0. However the immobilized enzyme showed higher activity than the free enzyme at pH 4.0, and in particular, rBGLa exhibited β-glucosidase activity under alkaline conditions above pH 8.0 when immobilized, even though it was not active in the free form (Fig. 3a). The results of pH stability testing indicated that immobilized rBGLa maintained its activity at approximately 100% of the original level after 2 h of incubation at the pH range of 5.0–6.0, whereas the free enzyme showed pH stability of 33–42%. Above pH 7, alginate beads were dissolved in the reaction buffer, which revealed that the immobilization carrier is unstable under these conditions.

The activity of immobilized rBGLa increased gradually with increasing temperature up to 50 °C, and the optimum temperature was shifted from 45 to 50 °C by immobilization (Fig. 3b). While the activity of free rBGLa decreased rapidly at 55 °C and was inactivated at 60 °C, the results indicated that the immobilized enzyme maintained more than 79% of its maximum activity at 55 and 60 °C. rBGLa was thermostable at 25–55 °C when immobilized, while the maximum thermal stability of free enzyme was about 60% at 25 °C. However, despite the presence of immobilized enzyme activity at 60 °C, it was completely inactivated after 2 h of incubation.

### Biotransformation of geniposide and hot-water extract of Gardeniae Fructus

**Fig. 4:**
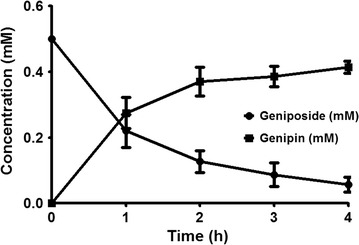
Time-course analysis of geniposide (*closed circle*) bioconversion into genipin (*closed square*) by rBGLa. The enzyme solution containing 0.5 μg/mL rBGLa was reacted with 0.5 mM geniposide in 25 mM sodium phosphate buffer (pH 6.0) at 45 °C. The reaction samples were withdrawn at regular intervals for 4 h and analyzed by HPLC

To investigate rBGLa activity on its substrate containing a β-d-glucopyranoside structure that has various effects on the human body, the enzyme activity was determined using a reaction with geniposide and Gardeniae Fructus, and then quantifying the concentrations of both geniposide and genipin by HPLC analysis. Before this analysis, we confirmed that geniposide and genipin in aqueous solution could be directly analyzed without organic solvent extraction as they exhibited solubility at 0.5 mM. The transformation efficiency of geniposide (0.5 mM) into its aglycone, genipin, at 0.5 μg/mL rBGLa was over 50% within 1 h, and 80% of the glucosidic linkages in geniposide were hydrolyzed in a 2-h reaction (Fig. 4).

**Fig. 5:**
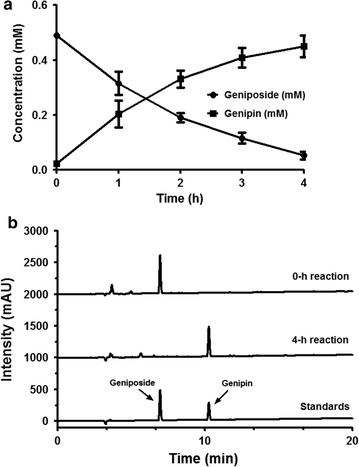
HPLC analysis for the bioconversion of Gardeniae Fructus hot-water extract using rBGLa. The enzymatic reaction of Gardeniae Fructus hot-water extract was performed in the reaction buffer at pH 6.0 and 45 °C. **a** Gardeniae Fructus extract (1.6 mg/mL) was reacted with free rBGLa (0.5 μg/mL), and the changes in geniposide and genipin concentration were monitored. **b** The transition of geniposide by immobilized rBGLa was monitored after 4-h reaction at 240 nm by HPLC, and was compared using standard geniposide and genipin

For the enzymatic conversion of a component of the herbal extract, Gardeniae Fructus containing geniposide as a functional marker compound was extracted in hot water at 115 °C, and the powder was prepared by filtering and freeze-drying the extract. Gardeniae Fructus extract was analyzed by HPLC, showing that 1.6 mg/mL extract contained 11.9% geniposide (w/w), which corresponds to approximately 0.5 mM, whereas genipin was present in negligible amounts (0.3%) (Fig. 5a). Through the enzymatic reaction of free rBGLa with Gardeniae Fructus extract, we confirmed that the geniposide in the extract at the same concentration as the single component reaction was also converted by reacting with 0.5 μg/mL rBGLa. The geniposide in the extract was completely hydrolyzed after a 4-h reaction, and the concentration of genipin was increased in a manner corresponding to the decrease of geniposide content, which is similar to the single geniposide reaction. Finally, we confirmed that the geniposide in the extract was also successfully converted into genipin by reacting with immobilized enzyme (Fig. 5b).

## Discussion

In this study, recombinant β-glucosidase from *L. antri* was expressed and purified after cloning of the gene into the pETDuet-1 vector to characterize the enzymatic properties and evaluate the efficiency of conversion of geniposide into its corresponding aglycone, genipin.

rBGLa linked with a (His)_6_-tag at the N-terminus was purified using immobilized metal affinity chromatography. Non-specific binding proteins were adequately removed by washing with 30 mM imidazole solution and rBGLa was finally eluted in the buffer containing 250 mM imidazole. To confirm the reaction conditions with the maximum activity of purified rBGLa, *p*NPG was used to analyze the enzyme characteristics, such as optimum temperature and pH. As a result, free rBGLa showed high activity in the range of 40–50 °C and the optimum temperature was 45 °C. In addition, the pH was found to be optimum at 6.0 and the activity decreased rapidly at pH levels other than 6.0. This suggests that β-glucosidase from *L. antri* also plays a role in digesting food containing plant-derived glycosides in the large intestine, similar to the colon, which is the part of the body where *Lactobacillus* commonly grows (Baati et al. 2000). As rBGLa was unstable upon changes in both temperature and pH, efforts to maintain the enzyme activity were required. To overcome its low stability, enzyme immobilization using an alginate carrier was carried out. The result showed that there was no pH shift in the maximum activity of the immobilized enzyme reported in previous studies (Arica et al. 1998; Erginer et al. 2000). However, the enzyme activity was improved at pH 4.0 and above 8.0 and the stability of the immobilized enzyme was highly improved at pH 5.0 and 6.0, suggesting that the alginate matrix protects the enzyme from structural denaturation by changes in pH (Keerti et al. 2014; Su et al. 2010). This result revealed that immobilization of rBGLa is effective at pH 5.0–6.0 despite the instability of the alginate carrier dissolved at a pH above 7.0 and enzyme activity at pH less than 4.0. The investigation of the effect of temperature on the immobilized enzyme indicated that the enzyme was active in the range of 25–60 °C and the optimal temperature of rBGLa was shifted to 50 °C when immobilized. In addition, enzyme stability was highly improved by immobilization, which maintained its original activity up to 55 °C after 2 h of incubation. These results may indicate that the alginate matrix preserves the three-dimensional structure of rBGLa despite a temperature increase to 55 °C (Keerti et al. 2014). The properties of immobilized rBGLa enable the conversion efficiency to be improved by preserving its activity and reuse of entrapped enzyme by simple recovery, which has a cost advantage (Mohamad et al. 2015; Sheldon 2007). Especially, for the bioconversion of food or drug substance enzyme immobilization is advantageous in eliminating the enzyme corresponding to contaminant in the final product after the reaction (Mohamad et al. 2015).

Geniposide is generally known to be metabolized into genipin by bacterial enzymes in the intestine or liver (Akao et al. 1994). In this study, we investigated whether geniposide can be effectively converted into genipin by purified rBGLa, which has biological activities including antioxidant, anti-inflammatory and anti-angiogenic effects. In the reaction of geniposide with rBGLa at 45 °C and pH 6.0, it was shown that genipin was generated upon a decrease in geniposide (at 0.4 mM, corresponding to 80% of the initial concentration), after 2 h of incubation. It demonstrated that β-glucosidase from *L. antri* can convert geniposide into genipin effectively by breaking the glucosidic linkage in the substrate.

Based on this result, we converted geniposide in plant extract using rBGLa. As an herb for enzymatic transformation, we selected Gardeniae Fructus, which contains a large quantity of geniposide as an index component for its quality control. HPLC analysis of the hot-water extract of Gardeniae Fructus showed that it contained a high level of geniposide, namely, 11.9% (w/w), while there was little genipin [0.3% (w/w)] in the extract in the previous report (Gong et al. 2014). As shown in Fig. 5a, 1.6 mg/mL Gardeniae Fructus contained approximately 0.5 mM geniposide, and the geniposide in the extract was hydrolyzed by free rBGLa to a similar pattern as single compound reaction (Fig. 4). The result may imply that there is no rBGLa inhibitors for geniposide conversion in the extract. This reveals that the rBGLa can also increase the genipin content by converting the geniposide present in herbal extracts, which contains various components, without enzyme inhibition. Based on these results, it may be expected that the use of herbal extracts containing geniposide transformed by the enzyme β-glucosidase from *L. antri* or fermented by the strain can improve the absorption rate and biological efficacy by direct action on the human body as the studies on bioconversion of various ginsenosides from ginseng using β-glucosidase (Hong et al. 2012; Quan et al. 2012). The reaction result of Gardeniae Fructus extract with immobilized rBGLa exhibited that the geniposide in the herb can be successfully converted into genipin by immobilized enzyme (Fig. 5b). In the industrial process for producing high content of genipin in the gardenia extract, enzymatic conversion is more advantageous when the enzyme was immobilized, which can improve the productivity by introducing continuous reaction system and reduce the production cost by reusing bio-catalyst and reacting the extract at a relatively higher temperature compared to optimum temperature of free enzyme after hot water extraction of Gardeniae Fructus, which saves energy consumption required for cooling the bioreactor (Chakraborty et al. 2016; Javed et al. 2016). We will investigate further the effect of increased aglycone content using rBGLa on various activities including antioxidative, anti-inflammatory, and antitumor effects.

## Abbreviations

rBGLa: recombinant β-glucosidase from *Lactobacillus antri*
*p*NPG: *p*-nitrophenyl β-d-glucopyranoside
*E. coli*: *Escherichia coli*
PCR: polymerase chain reaction
IPTG: isopropyl-β-d-thiogalactopyranoside
SDS-PAGE: sodium dodecyl sulfate polyacrylamide gel electrophoresis
*p*NP: *p*-nitrophenol
SDS: sodium dodecyl sulfate
HPLC: high-performance liquid chromatography
